# Dietary Inflammatory and Insulinemic Potentials, Plasma Metabolome and Risk of Colorectal Cancer

**DOI:** 10.3390/metabo13060744

**Published:** 2023-06-12

**Authors:** Dong Hoon Lee, Qi Jin, Ni Shi, Fenglei Wang, Alaina M. Bever, Jun Li, Liming Liang, Frank B. Hu, Mingyang Song, Oana A. Zeleznik, Xuehong Zhang, Amit Joshi, Kana Wu, Justin Y. Jeon, Jeffrey A. Meyerhardt, Andrew T. Chan, A. Heather Eliassen, Clary B. Clish, Steven K. Clinton, Edward L. Giovannucci, Fred K. Tabung

**Affiliations:** 1 Department of Sport Industry Studies, Yonsei University, Seoul 03722, Republic of Korea; dhlee@yonsei.ac.kr (D.H.L.);; 2 Department of Nutrition, Harvard T.H. Chan School of Public Health, Boston, MA 02115, USA; 3 Department of Exercise and Nutrition Sciences, Moyes College of Education, Weber State University, Ogden, UT 84408, USA; 4 Comprehensive Cancer Center, The Ohio State University, Columbus, OH 43210, USA; 5 Interdisciplinary Ph.D. Program in Nutrition, The Ohio State University, Columbus, OH 43210, USA; 6 Division of Medical Oncology, Department of Internal Medicine, College of Medicine, The Ohio State University, Columbus, OH 43210, USA; 7 Department of Epidemiology, Harvard T.H. Chan School of Public Health, Boston, MA 02115, USA; 8 Harvard-MIT Division of Health Sciences and Technology, Harvard Medical School, Boston, MA 02115, USA; 9 Department of Biostatistics, Harvard T.H. Chan School of Public Health, Boston, MA 02115, USA; 10 Channing Division of Network Medicine, Brigham and Women’s Hospital, Harvard Medical School, Boston, MA 02115, USA; 11 Clinical and Translational Epidemiology Unit, Massachusetts General Hospital, Harvard Medical School, Boston, MA 02115, USA; 12 Division of Gastroenterology, Massachusetts General Hospital, Harvard Medical School, Boston, MA 02115, USA; 13 Cancer Prevention Center, Yonsei Cancer Center, Seoul 03722, Republic of Korea; 14 Department of Medical Oncology, Dana-Farber Cancer Institute, Harvard Medical School, Boston, MA 02115, USA; 15 Broad Institute of MIT and Harvard, Cambridge, MA 02142, USA; 16 Department of Immunology and Infectious Diseases, Harvard T.H. Chan School of Public Health, Boston, MA 02115, USA; 17 Division of Epidemiology, College of Public Health, The Ohio State University, Columbus, OH 43220, USA

**Keywords:** empirical dietary inflammatory pattern, inflammation biomarkers, empirical dietary index for hyperinsulinemia, C-peptide, plasma metabolomic profiles, colorectal cancer

## Abstract

The inflammatory and insulinemic potentials of diets have been associated with colorectal cancer risk. However, it is unknown whether the plasma metabolite profiles related to inflammatory diets, or to insulinemic diets, underlie this association. The aim of this study was to evaluate the association between metabolomic profile scores related to the food-based empirical dietary inflammatory patterns (EDIP), the empirical dietary index for hyperinsulinemia (EDIH), and plasma inflammation (CRP, IL-6, TNFα-R2, adiponectin) and insulin (C-peptide) biomarkers, and colorectal cancer risk. Elastic net regression was used to derive three metabolomic profile scores for each dietary pattern among 6840 participants from the Nurses’ Health Study and Health Professionals Follow-up Study, and associations with CRC risk were examined using multivariable-adjusted logistic regression, in a case-control study of 524 matched pairs nested in both cohorts. Among 186 known metabolites, 27 were significantly associated with both the EDIP and inflammatory biomarkers, and 21 were significantly associated with both the EDIH and C-peptide. In men, odds ratios (ORs) of colorectal cancer, per 1 standard deviation (SD) increment in metabolomic score, were 1.91 (1.31–2.78) for the common EDIP and inflammatory-biomarker metabolome, 1.12 (0.78–1.60) for EDIP-only metabolome, and 1.65 (1.16–2.36) for the inflammatory-biomarkers-only metabolome. However, no association was found for EDIH-only, C-peptide-only, and the common metabolomic signatures in men. Moreover, the metabolomic signatures were not associated with colorectal cancer risk among women. Metabolomic profiles reflecting pro-inflammatory diets and inflammation biomarkers were associated with colorectal cancer risk in men, while no association was found in women. Larger studies are needed to confirm our findings.

## 1. Introduction

Colorectal cancer is a major cause of morbidity and mortality, in the United States and globally [1]. Several dietary patterns have shown associations with overall health [2,3,4], and with colorectal cancer prevention [5,6]. Some components of a Western-type diet, including a high intake of red and processed meats, refined carbohydrate-rich foods, added sugars, and a diet low in whole grains, fiber, fruits, and vegetables, are associated with an increased risk of colorectal cancer [5]. These findings are consistent across numerous populations, suggesting that some broad underlying mechanisms, influenced by overall dietary patterns, may be operative. Two factors of potential interest regarding cancer, as well as other chronic diseases, are inflammation and insulin resistance/hyperinsulinemia. Based on this premise, two empirical hypothesis-oriented dietary patterns have previously been developed and validated: (1) the empirical dietary inflammatory pattern (EDIP), to assess the potential of a diet to contribute to chronic systemic inflammation [7,8,9], and (2) the empirical dietary index for hyperinsulinemia (EDIH), to assess the potential of a diet to contribute to insulin resistance and the insulin response [10]. The EDIP was developed based upon weighting foods in relation to circulating inflammatory biomarkers including C-reactive protein (CRP), interleukin-6 (IL6), and tumor necrosis factor alpha receptor 2 (TNFα-R2); the EDIH was developed by weighting foods in relation to plasma C-peptide. Compared to other well-established dietary patterns (e.g., Dietary Approaches to Stop Hypertension (DASH), the Mediterranean dietary pattern, the alternative healthy eating index (AHEI), the healthy plant-based diet), the EDIP and EDIH were considerably more strongly associated with chronic disease risk (diabetes, cardiovascular diseases, obesity-related cancers) [4].

Furthermore, we found that the EDIP and EDIH are robustly associated with an increased risk of colorectal cancer, with a stronger association shown in men than in women [11,12,13,14]. Similarly, a positive association has been observed between C-peptide and colorectal cancer risk in nested case-control studies, especially in men [15], and Mendelian randomization studies have supported causal association between higher-fasting insulin (but not glucose traits or type 2 diabetes) and colorectal cancer risk [16]. Regarding inflammatory biomarkers, some meta-analyses of observational studies have found weak and inconsistent associations between circulating concentrations of CRP and IL6, and colorectal cancer risk [17,18,19], and Mendelian randomization studies in large multi-consortia have not supported these associations [20,21]. The observed inconsistent results, and some evidence of sex differences in the relationship between diet inflammatory and insulinemic potentials, and colorectal cancer risk, warranted further studies to elucidate the underlying biological mechanisms.

Metabolites are the final product of preceding molecular processes related to genomics, transcriptomics, and proteomics [22,23,24]. Metabolomics is thus uniquely suited to assess metabolic responses to diet. The objectives of the current study were to (1) identify metabolites associated with both the EDIP and plasma inflammatory biomarkers, and both the EDIH and plasma C-peptide, and (2) prospectively examine the association between these metabolomic signatures and the risk of colorectal cancer in a prospective nested case-control study. The hypothesis was that the metabolome associated with both the dietary pattern and corresponding biomarkers(s) may be more robustly predictive of colorectal cancer risk than the dietary-pattern-only metabolome.

## 2. Methods

### 2.1. Study Design and Population

The Nurses’ Health Study and Health Professionals Follow-up Study comprises ongoing prospective cohorts, with multiple factors, and numerous health outcomes. The Nurses’ Health Study was established in 1976, and the Health Professionals Follow-up Study started in 1986. The Nurses’ Health Study (*n* = 121,701) enrolled female-registered nurses (married at the time) aged 30–55 years in 1976 [25]. The Health Professionals Follow-up Study (*n* = 51,529) enrolled male health professionals (dentists, veterinarians, optometrists, osteopathic physicians, pharmacists, and podiatrists) aged 40–75 years in 1986. Blood samples were collected from subsamples of the Nurses’ Health Study (*n* = 32,826) from 1989 to 1990, and the Health Professionals Follow-up Study (*n* = 18,225) from 1993 to 1994, and were archived below −130 degrees Celsius [26,27]. The participants were sent follow-up questionnaires to update data and to identify newly diagnosed diseases, including colorectal cancer. The response rate to each questionnaire exceeded 90% over the follow up period.

**Figure 1:**
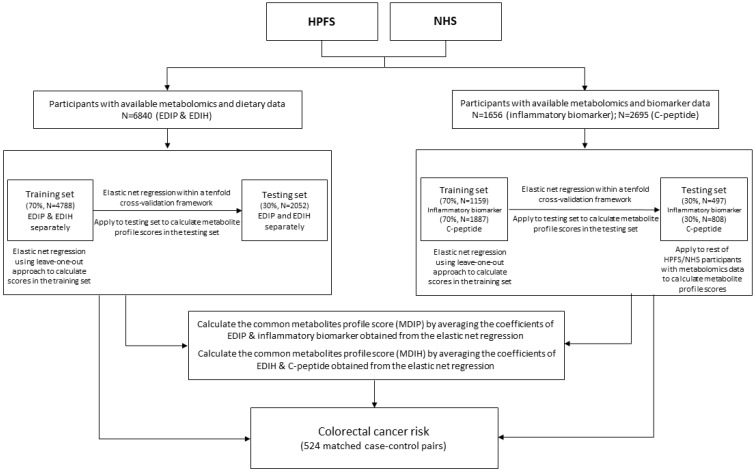
Flow chart describing the study sample and study design. EDIH is the empirical dietary index for hyperinsulinemia; EDIP is the empirical dietary inflammatory potential; HPFS is the Health Professionals Follow-up Study; MDIH is the metabolomic dietary index for hyperinsulinemia; MDIP is the metabolomic dietary inflammatory potential; NHS is the Nurses’ Health Study.

In this report, metabolomic data from previously matched nested case-control studies using a variety of endpoints, nested within each of these two cohorts for a total of 6840 (6143 women and 697 men), were used. This sample included a subsample of 616 women (Nurses’ Health Study), and 546 men (Health Professionals Follow-up Study), with the colorectal cancer case-control data followed from blood collection until 2014 for newly diagnosed colorectal cancer (524 colorectal cancer cases, and 524 controls). We first used the entire dataset to define the metabolomic patterns, and then examined these in relation to colorectal cancer risk in the nested case-control data set. A flowchart of the study design and sample is presented in Figure 1.

The study protocol was approved by the institutional review boards of the Brigham and Women’s Hospital, and Harvard T.H. Chan School of Public Health, and those of participating registries as required. Informed consent was obtained from all subjects involved in the study.

### 2.2. Dietary Assessment

Dietary information was collected through a self-administered validated semi-quantitative food frequency questionnaire (FFQ) up to the time of the blood draw in each cohort as follows: 1984, 1986, and 1990 in the Nurses’ Health Study; 1986, 1990, and 1994 in the Health Professionals Follow-up Study. The FFQs assessed habitual intake of foods and beverages over the preceding year, and participants reported how often, on average, they consumed each food of a specified standard portion size [28,29,30,31,32]. These questionnaires have been extensively validated through comparison to detailed dietary records, to assess intake of various nutrients, individual foods, food groups, and dietary patterns [28,29,32,33,34].

The EDIP and EDIH scores were previously developed in the Nurses’ Health Study, and validated in the Nurses’ Health Study II and the Health Professionals Follow-up Study [9]. Thirty-nine pre-defined food groups were included, in reduced rank regression models followed by stepwise linear regression analyses, to identify a dietary pattern most predictive of important inflammatory biomarkers, specifically CRP, IL6, and TNFα-R2. The EDIP score was a weighted sum of 18 food groups (processed meat, red meat, organ meat, other fish, other vegetables, refined grains, high-energy beverages, low-energy beverages (e.g., diet sodas), and tomatoes contributed to higher or more pro-inflammatory scores, whereas beer, wine, tea, coffee, dark yellow vegetables, leafy green vegetables, snacks (e.g., popcorn), fruit juice, and pizza contributed to lower or more anti-inflammatory scores) [9]. Similarly, 39 pre-defined food groups were included in the stepwise linear regression analyses, to identify a dietary pattern most predictive of C-peptide [10]. The EDIH score was a weighted sum of 18 food groups (red meat, low-energy beverages, cream soups, processed meat, margarine, poultry, butter, French fries, other fish, high-energy beverages, tomatoes, low-fat dairy products, and eggs contributed to higher or more hyperinsulinemic diets, whereas wine, coffee, whole fruits, high-fat dairy products, and green-leafy vegetables contributed to lower or more low insulinemic diets) [10]. Nine of the food groups overlapped between the two dietary patterns, and each had nine additional unique contributors (Supplementary Tables S1 and S2).

For this study, the dietary data closest to the blood draw were used to calculate the EDIP and EDIH scores. These were from FFQs collected in 1990 and 1994 for the Health Professionals Follow-up Study, 1986 and 1990 for the Nurses’ Health Study, and 1995 and 1999 for the Nurses’ Health Study-II. Using the average dietary data from these two questionnaires helped to reduce within-person variation, and best represent long-term diet. Participants with excessive missing items (≥70) on the FFQs, or implausibly low or high energy intake (<600 or >3500 kcal/d for women and <800 or >4200 kcal/d for men), were excluded [35].

### 2.3. Covariate Assessment

Information on covariates such as age, race/ethnicity, height, weight, menopausal status, physical activity, regular aspirin use, smoking status, family history of disease, screening, and alcohol intake, was collected from all three cohorts using biennial questionnaires. Participants reported smoking status (classified as never, former, or current). Participants’ body mass index (BMI, kg/m^2^) was calculated using height (meters) reported at baseline for each cohort, and weight (kilograms) reported at each biennial questionnaire cycle. Physical activity was calculated by summing the mean metabolic equivalent task hours per week for each reported physical activity, which included tennis/squash/racquetball, rowing, calisthenics, walking, jogging, running, bicycling, and swimming. The physical activity was then expressed in metabolic equivalent task hours per week. Regular use of aspirin or other nonsteroidal anti-inflammatory drugs (NSAIDs) was defined as the use of ≥2 standard tablets (325 mg) of aspirin or ≥2 tablets of NSAIDs/week.

### 2.4. Plasma Inflammatory Biomarker Assessment and Metabolomics Profiling

The procedures for the measurement of plasma CRP, IL6, TNFα-R2, adiponectin, and C-peptide have been previously described [36]. In brief, IL-6 and TNFα-R2 concentrations were measured using ELISAs (R&D Systems). CRP was measured using a high-sensitivity immunoturbidimetric assay (Denka Seiken). Adiponectin concentrations were measured using a competitive radioimmunoassay (Linco Research). The intra-assay coefficient of variation (CV) from blinded quality-control (QC) samples ranged from 1.0% to 9.1% for CRP, 2.9% to 12.8% for IL6, 4.0% to 10.0% for TNFα-R2, and 8.1% to 11.1% for adiponectin across batches. C-peptide was measured by ELISA (Diagnostic Systems Laboratories/Beckman Coulter). The average intra-assay coefficient of variation in blinded quality-control samples was <12% across batches. We had previously evaluated the stability of biomarkers in whole blood for 24 to 48 h [37]. Blinded QC samples were randomly interspersed and included in the laboratory analyses. Data were available for all four biomarkers among 1656 participants, and for C-peptide among 2695 participants.

Plasma metabolites were measured as peak areas using a targeted liquid chromatography–tandem mass spectrometry (LC-MS/MS) metabolomics platform at the Broad Institute (Cambridge, MA, USA). Metabolite-profiling methods were developed using reference standards of metabolites, to determine chromatographic retention times, MS multiple reaction monitoring transitions, declustering potentials, and collision energies [38,39,40,41]. For metabolites that were missing in <10% of the sample, half of the sample minimum was imputed. A total of 186 metabolites were available in both cohorts. Participants were cancer-free when they provided blood samples, and had similar general demographic and lifestyle factors compared with the overall cohort.

### 2.5. Colorectal Cancer Assessment

For the Nurses’ Health Study and Health Professionals Follow-up Study, participants were asked, through biennial questionnaires, whether they were diagnosed with colorectal cancer or other specified diseases. When a participant reported a diagnosis of colorectal cancer, the participant’s permission would be obtained to review their medical records and pathology reports. Physicians reviewed self-reported cases of colorectal cancer based on the medical records and pathology reports, while blinded to exposure information and abstracted information on histological type, anatomic location, and cancer stage. Proximal colon cancers were defined if they occurred in the cecum, ascending colon, or transverse colon; distal colon cancers if they occurred in the descending colon or sigmoid colon; and rectal cancers if they occurred in the rectosigmoid junction or rectum. Our study only included colorectal cancer cases that were confirmed through the medical record review.

### 2.6. Statistical Analysis

Before the analyses, all 186 named metabolites were transformed using probit scores to improve normality, and using z-scores for standardization to improve between-metabolite comparability. An overall biomarker z-score was created, combining the z-scores for all four biomarkers (i.e., zcrp + zil6 + ztnfar2 − zadiponectin). To identify the metabolomic signatures, all participants were randomly assigned to a training set (70%) and testing set (30%) (Figure 1) [42,43]. In the training set, EDIP, overall inflammatory biomarker z-score, EDIH, or C-peptide was regressed on the 186 metabolites, using elastic net regression with 10-fold cross-validation [44]. Then, the trained model was applied to the testing set, to calculate the metabolomic profile scores as weighted sums of the selected metabolites with weights equal to beta coefficients from the elastic net regression. The leave-one-out cross-validation approach was used to calculate the metabolomic signatures in the training set, and avoid overfitting the data. To create the metabolomic dietary inflammatory pattern (MDIP) score; that is, a metabolomic profile score reflecting the metabolic potential of both an inflammatory dietary pattern and circulating inflammatory biomarkers; the coefficients of the EDIP and inflammatory biomarker z-scores obtained from the elastic net regressions were averaged. The same procedure was conducted to identify metabolites associated with both the EDIH and C-peptide (Figure 1).

Three EDIP-related metabolomic signatures were created: (1) a metabolomic profile score including metabolites associated mainly with the EDIP, irrespective of their association with biomarkers (EDIP-only metabolome), (2) a metabolomic profile score comprised of metabolites associated mainly with inflammatory biomarkers, not considering their associations with the EDIP (BIOM-only metabolome), and (3) a metabolomic profile score reflecting the metabolic potential of both an inflammatory dietary pattern, and circulating inflammatory biomarkers (metabolomic dietary inflammatory pattern (MDIP)). Similarly, three EDIH-related metabolomic signatures were created: (1) a metabolomic profile score including metabolites associated mainly with the EDIH, irrespective of their association with biomarkers (EDIH-only metabolome), (2) a metabolomic profile score comprised of metabolites associated mainly with C-peptide, not considering their associations with the EDIH (CPEP-only metabolome), and (3) a metabolomic profile score reflecting the metabolic potential of both an insulinemic dietary pattern, and circulating C-peptide (metabolomic dietary index for hyperinsulinemia (MDIH)).

Next, the associations between the EDIP (diet), the EDIH (diet), and the six metabolomic profile scores (MDIP, EDIP-only, BIOM-only, MDIH, EDIH-only, and CPEP-only) in 1 standard deviation (SD) increment, and colorectal cancer risk, were examined, separately by sex, using conditional logistic regression analyses. Basic models were adjusted for only the matching factors, including age (continuous), sex (male or female), time of blood collection (continuous), race/ethnicity (white or non-white), and menopausal status for women (premenopausal, postmenopausal, or postmenopausal hormone use). The multivariable model was further adjusted for BMI (continuous), physical activity (continuous), regular aspirin use (yes or no), smoking status (never, past, current smoker), family history of colorectal cancer (yes or no), endoscopy (yes or no), alcohol intake (continuous), and total calorie intake (continuous). An additional multivariable model further adjusting for coffee intake (which contribute to a lower EDIP and EDIH), but which had metabolites related to caffeine, which may not influence inflammation, was conducted. To gain insight into potential metabolic pathways, individual metabolites of MDIP and MDIH were examined in relation to colorectal cancer risk. All analyses were performed using an R package for metabolomic signature generation, and SAS version 9.4 (SAS Institute Inc, Cary, NC, USA) for all other analyses.

## 3. Results

### 3.1. Participant Characteristics and Identification of Metabolomic Signatures

Selected participant characteristics, in each of the two cohorts, and by colorectal cancer case-control status in the colorectal cancer subsample, are summarized in Table 1. The mean age and BMI were 60 years and 25 kg/m^2^, respectively. The majority of participants were White. There were no differences in characteristics among the participants for the metabolomic, and colorectal cancer case-control study, analyses. Compared with the controls, the cases tended to be more likely to be overweight or obese, not use NSAIDs, have a positive family history of colorectal cancer, and be less likely to have had a previous lower gastrointestinal endoscopy.

Among 186 known metabolites, 82 metabolites for the EDIP, 43 for the inflammatory biomarker z-score, 120 for the EDIH, and 25 for C-peptide from the elastic net regressions were identified. A total of 27 metabolites were commonly associated with both the EDIP and the inflammatory biomarker z-score, and 21 metabolites were commonly associated with both the EDIH and C-peptide. The EDIP (diet) had correlations of 0.42 with EDIP-only, 0.21 with BIOM-only, and 0.20 with MDIP metabolomic scores. The EDIH (diet) had correlations of 0.44 with EDIH-only, 0.14 with CPEP-only, and 0.15 with MDIH metabolomic scores.

### 3.2. Association between Metabolomic Profile Scores and Colorectal Cancer Risk

Metabolomic profiles based on both pro-inflammatory diets and inflammation biomarkers were associated with colorectal cancer risk in men (Table 2). The ORs per 1 SD increment (and 95% CIs) were 1.91 (1.31–2.78) for MDIP, 1.12 (0.78–1.60) for EDIP-only, and 1.65 (1.16–2.36) for inflammatory-biomarkers-only metabolomic scores. Similarly, the EDIP (diet) was significantly associated with colorectal cancer risk in men in the nested case-control study (OR = 1.47, 95% CI: 1.03–2.09), and in the full cohort (OR = 1.17, 95% CI: 1.09–1.26). The results for the EDIP (diet) in the nested case-control study strengthened substantially when adjusted for coffee, though the adjustment for coffee had only a minor effect in the entire study population. However, no association was found between EDIP-related metabolomic signatures and colorectal cancer risk in women (Table 2). The ORs per 1 SD increment were 1.00 (0.85–1.19) for MDIP, 1.13 (0.94–1.36) for EDIP-only, and 1.05 (0.89–1.24) for inflammatory-biomarkers-only metabolomic scores in women. Similarly, the EDIP (diet) was not significantly associated with colorectal cancer risk in women (OR = 1.24, 95% CI: 0.88–1.76). However, the association was statistically significant when the EDIP (diet) was analyzed in the full cohort in women (OR: 1.08; 95% CI: 1.02–1.16).

Metabolomic profiles reflecting insulinemic diets and the C-peptide biomarker were not significantly associated with colorectal cancer risk (Table 3). In men, the ORs per 1 SD increment were 1.12 (0.82–1.52) for MDIH, 0.97 (0.70–1.34) for EDIH-only, and 1.26 (0.90–1.77) for C-peptide-only metabolomic scores. In women, the ORs per 1 SD increment were 0.95 (0.81–1.12) for MDIH, 1.20 (1.01–1.42) for EDIH-only, and 0.97 (0.82–1.14) for C-peptide-only metabolomic scores. However, the EDIH (diet) was significantly associated with colorectal cancer risk in men and women in the full cohort, and a similar pattern was shown in the nested case-control analysis, though the association was not statistically significant in men.

When the individual associations between the common metabolites of the EDIP and inflammatory biomarkers, and colorectal cancer risk, were examined (Supplementary Table S1) in men, positive associations were found for 1-methylguanosine (OR: 1.82; 95% CI: 1.26–2.62), N2.N2-dimethylguanosine (OR: 1.57; 95% CI: 1.08–2.29), and N-carbamoyl-beta-alanine (OR: 1.43; 95% CI: 1.04–1.97), while an inverse association was found for C36:2 PE plasmalogen (OR: 0.72; 95% CI: 0.52–0.99). On the other hand, C2 carnitine (OR: 1.24; 95% CI: 1.05–1.47), and biliverdin (OR: 1.21; 95% CI: 1.04–1.41) were significantly positively associated in women. Among the individual metabolites commonly associated with the EDIH and C-peptide, C38:2 PE (OR: 1.43; 95% CI: 1.04–1.97) was positively associated in men, while C3 carnitine (OR: 1.18; 95% CI: 1.00–1.38) was positively associated with colorectal cancer risk in women (Supplementary Table S2).

## 4. Discussion

In the current study, an integrated analysis of the association between inflammatory and insulinemic dietary patterns, plasma inflammation, and insulin biomarkers, plasma metabolomics, and colorectal cancer risk, was conducted. We identified 27 metabolites associated with both an inflammatory dietary pattern, and plasma inflammation biomarkers, termed the common metabolome for inflammation (MDIP); and 21 metabolites associated with both an insulinemic dietary pattern, and C-peptide levels, termed the common metabolome for hyperinsulinemia (MDIH). When we examined the association between the identified metabolomic signatures, and colorectal cancer risk in a nested case-control study, metabolomic profiles reflecting pro-inflammatory diets and inflammation biomarkers were associated with colorectal cancer risk in men. However, we found no association for metabolomic profiles reflecting insulinemic diets (MDIH) and C-peptide in men. Moreover, the metabolomic signatures for insulin and inflammation were not associated with colorectal cancer risk among women.

As reported earlier, we did observe associations with increased risk for EDIP and EDIH among both men and women in the full cohort. Consistent with previous studies, we found that the EDIP and EDIH were positively associated with colorectal cancer risk in a nested case-control study with metabolomic data, though confidence intervals were wide (especially in men), given the much-smaller sample sizes. For men, signatures for MDIP (robustly) and MDIH (suggestively), and biomarker-only, were associated with the risk of colorectal cancer. However, these metabolomic patterns were not associated with colorectal cancer in women. The limited sample size for the metabolomic analyses, resulting in statistical instability, may have played a role in the inconsistent results, though the number of case-control pairs was greater in women (*n* = 394) than in men (*n* = 130). Thus, sample size alone is not likely to explain the sex differences. In fact, we had lower power in men than in women to detect associations.

The weak association between MDIH and colorectal cancer risk may be surprising, given previous support for the insulin–colorectal-cancer hypothesis, especially in men. Although insulin resistance and hyperinsulinemia are related, their relationship varies over time. At earlier stages in the natural history of insulin resistance and diabetes, insulin resistance strongly determines hyperinsulinemia. Over time, as beta-cells deplete, insulin resistance may remain high, but insulin levels lower. Over a longer time course, there may be a hypoinsulinemic response (especially for post-prandial insulin). It is possible that metabolomics may be more closely associated with insulin resistance than with high levels of insulin (especially in the fasting state), although we could not directly evaluate this speculation. If a circulating level of insulin (a mitogen and anti-apoptotic hormone) is the direct causal factor, as suggested by a Mendelian randomization study [16], then direct measures of insulin or C-peptide might be expected to yield more robust associations than studies of associated metabolites. Future studies should assess the potential differential roles of insulin resistance and hyperinsulinemia in relation to colorectal cancer.

Larger studies are required to confirm if the stronger associations we saw in men are robust, or due to random fluctuation resulting from a limited sample size. Some data do suggest that the association between insulin resistance and colorectal cancer is stronger in men than in women [15]. In addition, high body mass index, the dominant determinant of inflammation and insulin resistance, has been a stronger risk factor for colorectal cancer in men than in women in most studies [45]. Of note, we had found that the EDIH was a strong risk factor in younger (mostly pre-menopausal) women in the Nurses’ Health Study 2 cohort, [46] while the current biomarker study was based in mostly post-menopausal women. Thus, it would be important to examine these associations in younger cohorts of women. Differences in obesity-related factors due to sex and age have been speculated upon, but no clear mechanisms have been established [45].

We observed evidence of sex differences in the relationship between metabolomic signatures of an inflammatory dietary pattern and biomarkers, and colorectal cancer risk. A significant positive association was found only in men, and not in women. When we examined the individual metabolites for MDIP with colorectal cancer, we also found differences in the metabolites associated with colorectal cancer risk by sex. In men, 1-methylguanosine, N2.N2-dimethylguanosine, and N-carbamoyl-beta-alanine were positively associated, and C36:2 PE plasmalogen was inversely associated, with colorectal cancer risk, but C2 carnitine and biliverdin were positively associated with colorectal cancer risk in women. These findings suggest potentially different biological mechanisms linking dietary inflammatory potential to colorectal cancer development by sex, or may reflect random variation. Several modified nucleosides in urine, including 1-methylguanosine and N2.N2-dimethylguanosine, have been identified since the mid-1980s as biochemical indicators of malignant disease [47], including colorectal cancer [48], yet their sex-based differences have not been elucidated. Even though metabolic reprogramming is an essential process in carcinogenesis, sex differences in cancer metabolism have not been widely considered [49]. It has been suggested that men exhibit higher rates of glucose and amino-acid utilization, whereas women favor lipid substrates for energy metabolism [49]. In addition, acylcarnitines, including C2-carnitine and C3-carnitine, found in the current study to be associated with higher colorectal cancer risk among women, have been associated with multiple gynecological cancers including ovarian cancer [50] and endometrial cancer [51]. Our findings are in line with the previous literature showing that compared to women, men have a higher exposure to environmental risk factors, and are also potentially more susceptible to such risk factors, including obesity, physical inactivity, alcohol, smoking, and poor diets [52].

There were a number of strengths to our study design. The sample for the derivation of metabolomic profile scores was sufficiently large to produce reliable patterns. We utilized a robust statistical methodology to identify the important metabolites. Elastic net regression has the advantage of effectively dealing with highly correlated variables (e.g., metabolites), while maintaining the quality of model selection. Our cohorts contained comprehensive covariate data, which allowed the control of potential confounding factors. We used state-of-the art interdisciplinary techniques to integrate metabolomic, biomarker, and dietary data, to identify potential mechanistic pathways linking dietary patterns and colorectal cancer risk. Finally, the repeated measuring of diet reduced potential measurement error.

However, our study had several limitations. Firstly, we had a relatively small colorectal cancer nested sample (524 case-control pairs), which may have reduced the statistical power to detect significant differences. Of relevance to this, similar-sized but statistically significant associations for diet variables were observed in the full cohort, indicating that our power may have been limited in the nested case-control study. Although important on a population basis, the relative risks were relatively moderate in magnitude. Secondly, metabolomic measurement was conducted once for each participant. However, our previous study showed high stability for most of the metabolites over 1 to 2 years within individuals. Of note, this approach in our cohort did produce robust findings for both insulin and inflammation metabolites with risk of type 2 diabetes, a disease that is strongly associated with these biological processes. Colorectal cancer has multiple etiologic factors, of which insulin resistance and inflammation may be one of many, so associations would be expected to be weaker than for diabetes. It is also possible that associations are stronger for molecular or anatomical sub-types of colorectal cancer, but we could not examine this with adequate power [53]. Moreover, the current study collected samples and information in the 1990s, and thus the findings may not accurately represent current environmental, feeding, food-processing, and human-behavior conditions. Lastly, participants in our study were predominantly White health professionals, which may limit the generalizability of the findings. However, the results for multiple exposures and various cancers from these cohorts were highly concordant with the literature in general [54].

In conclusion, we found that metabolomic profiles reflecting pro-inflammatory diets and inflammation biomarkers were associated with colorectal cancer risk in men. Results for hyperinsulinemic profiles were suggestive in men. However, no association was observed between the metabolomic signatures and colorectal cancer risk in women. Larger studies are needed to confirm our findings.

## Figures and Tables

**Table 1:** Characteristics of study participants.

	Metabolomic Studies		CRC Nested Case-Control Study	
	HPFS (*n* = 697)	NHS (*n* = 6143)	Control (*n* = 524)	Case (*n* = 524)
Age, year	63.3 (8.1)	57.4 (6.8)	60.2 (7.6)	60.2 (7.6)
BMI, kg/m^2^				
Lean	58	56	54	49
Overweight	37	30	32	37
Obese	5	14	14	15
Physical activity, MET-h/week	35.6 (32.4)	15.2 (10.1)	19.5 (20.7)	20.0 (22.3)
Total calorie, kcal/day	2016 (567)	1775 (465)	1853 (511)	1854 (523)
Women, %	0	100	75.2	75.2
White, %	96	96	99	97
NSAIDs use, %	46	25	33	29
Smoking, %				
Never	48	46	43	43
Past	47	41	45	43
Current	5	13	12	14
Menopausal status, %				
Missing/dubious	NA	10	8	8
Premenopausal	NA	17	14	15
Post on PMH	NA	33	31	28
Post but not on PMH	NA	40	47	49
Family history of CRC, %	15	13	14	17
Endoscopy, %	33	38	37	33

Values are means (SDs) for continuous variables, and percentages for categorical variables. CRC is colorectal cancer; HPFS is Health Professionals Follow-up Study; MET is metabolic equivalent task; NHS is Nurses’ Health Study; PMH is postmenopausal hormone therapy.

**Table 2:** Associations between the inflammatory dietary pattern, and the metabolomic potential of an inflammatory dietary pattern, and colorectal cancer risk, stratified by sex.

OR (95% CI)	Men		Women	
	Per 1 SD Increase in Score	*p*-Value	Per 1 SD Increase in Score	*p*-Value
MDIP signature				
Case/control	130/130		394/394	
Basic model	1.58 (1.15–2.17)	0.005	1.02 (0.89–1.18)	0.78
MV model 1	1.89 (1.30–2.74)	<0.001	0.99 (0.84–1.17)	0.87
MV model 2	1.91 (1.31–2.78)	<0.001	1.00 (0.85–1.19)	0.97
EDIP-only signature				
Case/control	130/130		394/394	
Basic model	0.97 (0.75–1.26)	0.83	1.06 (0.92–1.23)	0.40
MV model 1	1.00 (0.74–1.36)	0.99	1.05 (0.89–1.24)	0.56
MV model 2	1.12 (0.78–1.60)	0.55	1.13 (0.94–1.36)	0.18
BIOM-only signature				
Case/control	130/130		394/394	
Basic model	1.37 (1.02–1.84)	0.04	1.06 (0.92–1.22)	0.65
MV model 1	1.60 (1.13–2.27)	0.008	1.04 (0.88–1.23)	0.69
MV model 2	1.65 (1.16–2.36)	0.006	1.05 (0.89–1.24)	0.57
EDIP score (diet)				
Case/control	130/130		394/394	
Basic model	0.99 (0.80–1.24)	0.95	1.08 (0.93–1.24)	0.31
MV model 1	1.20 (0.90–1.61)	0.21	1.04 (0.89–1.22)	0.63
MV model 2	1.47 (1.03–2.09)	0.03	1.12 (0.94–1.34)	0.21
EDIP score (diet)—full cohort ^a^				
Case/person-year	1189/968,564		1486/1,685,241	
Basic model	1.10 (1.03–1.17)	0.003	1.05 (0.99–1.11)	0.08
MV model 1	1.15 (1.08–1.23)	<0.001	1.06 (1.01–1.13)	0.03
MV model 2	1.17 (1.09–1.26)	<0.001	1.08 (1.02–1.16)	0.02

Basic models were conducted using conditional logistic regressions that included the matching factors only (age, time of blood collection, race/ethnicity, and menopausal status for women). Multivariable (MV) model 1 was further adjusted for BMI, physical activity, regular aspirin use, smoking status, family history of colorectal cancer, endoscopy, alcohol intake, and total calorie intake. MV model 2 was further adjusted for coffee intake. ^a^ Analyses using all participants (regardless of metabolomic data) from Nurses’ Health Study and Health Professionals Follow-up Study. BIOM is inflammatory biomarkers; EDIP is the empirical dietary inflammatory potential; MDIP is the metabolomic dietary inflammatory potential.

**Table 3:** Associations between the insulinemic dietary pattern, and the metabolomic potential of an insulinemic dietary pattern, and colorectal cancer risk, stratified by sex.

OR (95% CI)	Men		Women	
	Per 1 SD Increase in Score	*p*-value	Per 1 SD Increase in Score	*p*-value
MDIH signature				
Case/control	130/130		394/394	
Basic model	1.12 (0.86–1.45)	0.40	0.97 (0.84–1.13)	0.72
MV model 1	1.11 (0.82–1.51)	0.48	0.95 (0.80–1.11)	0.50
MV model 2	1.12 (0.82–1.52)	0.48	0.95 (0.81–1.12)	0.55
EDIH-only signature				
Case/control	130/130		394/394	
Basic model	0.95 (0.73–1.23)	0.69	1.14 (0.99–1.32)	0.06
MV model 1	0.92 (0.68–1.25)	0.61	1.15 (0.98–1.36)	0.09
MV model 2	0.97 (0.70–1.34)	0.85	1.20 (1.01–1.42)	0.04
CPEP-only signature				
Case/control	130/130		394/394	
Basic model	1.20 (0.90–1.59)	0.21	0.98 (0.85–1.13)	0.80
MV model 1	1.25 (0.89–1.75)	0.19	0.95 (0.81–1.12)	0.55
MV model 2	1.26 (0.90–1.77)	0.19	0.97 (0.82–1.14)	0.66
EDIH score (diet)				
Case/control	130/130		394/394	
Basic model	1.04 (0.82–1.32)	0.76	1.21 (1.05–1.40)	0.01
MV model 1	1.13 (0.85–1.51)	0.40	1.22 (1.03–1.43)	0.02
MV model 2	1.20 (0.89–1.63)	0.24	1.27 (1.07–1.50)	0.006
EDIH score (diet)—full cohort ^a^				
Case/person-year	1189/968,564		1486/1,685,241	
Basic model	1.10 (1.03–1.17)	0.007	1.08 (1.01–1.15)	0.02
MV model 1	1.13 (1.06–1.22)	<0.001	1.07 (1.00–1.14)	0.04
MV model 2	1.13 (1.06–1.22)	<0.001	1.08 (1.01–1.15)	0.03

Basic models were conducted using conditional logistic regressions that included the matching factors only (age, sex, time of blood collection, race/ethnicity, and menopausal status for women). Multivariable (MV) model 1, further adjusted for BMI, physical activity, regular aspirin use, smoking status, family history of colorectal cancer, endoscopy, alcohol intake, and total calorie intake. MV model 2, further adjusted for coffee intake. ^a^ Analyses using all participants (regardless of metabolomic data) from Nurses’ Health Study and Health Professionals Follow-up Study. CPEP is C-peptide; EDIH is the empirical dietary index for hyperinsulinemia; MDIH is the metabolomic dietary index for hyperinsulinemia.

## Data Availability

The datasets generated and analyzed during the current study are available from the corresponding author on reasonable request. Data is not publicly available due to privacy or ethical restrictions.

## Supplementary Materials

The following supporting information can be downloaded at: https://www.mdpi.com/article/10.3390/metabo13060744/s1, Table S1. The food components of the empirical dietary inflammatory pattern (EDIP); Table S2. The food components of the empirical dietary index for hyperinsulinemia (EDIH); Table S3. Association of common metabolites of EDIP and inflammatory biomarker z-score with colorectal cancer risk; Table S4. Association of common metabolites of EDIH and C-peptide biomarker with colorectal cancer risk.
