# Supplementary material for: Dietary Inflammatory and Insulinemic Potentials, Plasma Metabolome and Risk of Colorectal Cancer
Source: Metabolites. 2023 Jun 12;13(6):744. doi: 10.3390/metabo13060744 (PMC10304271; doi:10.3390/metabo13060744)
Supplement: Supplementary file 1 [file metabolites-13-00744-s001.zip › metabolites-2395992-supplementary.pdf]

Supplementary materials

Supplementary Table S1. The food components of the empirical dietary inflammatory pattern (EDIP)

EDIP components	Food item
Positive association	
Processed meat	Processed meats, bacon, hot dog
Red meat	Beef, pork, lamb and hamburger patty
Organ meat	Beef, calf, pork, chicken and turkey livers
Other fish	Canned tuna, shrimp, lobster, scallops, fish, and other seafood other than dark meat fish
Other vegetables	Stick celery, mushrooms, pepper, corn, mixed vegetables, eggplant, zucchini, alfalfa sprouts and cucumber
Refined grain	White bread, English muffin, bagel, roll, muffin, biscuit, white rice, pasta, pancakes and waffles
High-energy beverage	Cola with sugar, other carbonated beverages with sugar and fruit punch drinks
Low-energy beverage	Low-energy cola, other low-energy carbonated beverages
Tomato	Fresh tomato, tomato juice, tomato sauce
Inverse association	
Beer	Beer
Wine	Red or white wine
Tea	Tea (not herbal)
Coffee	Coffee, decaffeinated coffee
Dark yellow vegetable	Carrots, yellow squash, yams and sweet potatoes
Green leafy vegetable	Spinach, iceberg or head lettuce, romaine and leaf lettuce
Snack	Potato or corn chips, popcorn and crackers
Fruit juice	Apple juice or cider, orange juice, grapefruit juice and other juice
Pizza	Pizza

Supplementary Table S2. The food components of the empirical dietary index for hyperinsulinemia (EDIH)

EDIH components	Food item
Positive association	
Red meat	Beef, pork, lamb and hamburger patty
Low-energy beverage	Low-energy cola, other low-energy carbonated beverages
Cream soup	Chowder or cream soup
Processed meat	Processed meats, bacon, hot dog
Margarine	Margarine
Poultry	Chicken, turkey
Butter	Butter
French fries	French fries
Other fish	Canned tuna, shrimp, lobster, scallops, fish, and other seafood other than dark meat fish
High-energy beverage	Cola with sugar, other carbonated beverages with sugar and fruit punch drinks
Tomato	Fresh tomato, tomato juice, tomato sauce
Low-fat dairy products	Skimmed or low-fat milk, sherbet or ice milk, yogurt
Eggs	Egg
Inverse association	
Wine	Red or white wine
Coffee	Coffee, decaffeinated coffee
Whole fruits	Raisins, grapes, avocado, banana, cantaloupe, watermelon, apple, pear, orange, grapefruit, strawberries, blueberries, peaches, apricots, plums
High-fat dairy products	Whole milk, cream, sour cream, ice cream, cream cheese, other cheese
Green leafy vegetable	Spinach, iceberg or head lettuce, romaine and leaf lettuce

Supplementary Table S3. Association of common metabolites of EDIP and inflammatory biomarker z-score with colorectal cancer risk

HMDB ID	Metabolites	Subclass	HR per 1-SD All participants	HR per 1-SD Men	HR per 1-SD Women
HMDB0000201	C2 carnitine	Fatty acid esters	**1.18 (1.03-1.36)**	1.06 (0.81-1.40)	**1.24 (1.05-1.47)**
HMDB0001008	Biliverdin	Bilirubins	**1.16 (1.01-1.33)**	1.02 (0.75-1.37)	**1.21 (1.04-1.41)**
HMDB0001563	1-methylguanosine	Nucleosides, nucleotides, and analogues	**1.16 (1.00-1.33)**	**1.82 (1.26-2.62)**	1.04 (0.88-1.22)
HMDB0001276	N-acetylspermidine	Carboximidic acids	1.14 (0.99-1.30)	1.31 (0.94-1.84)	1.10 (0.94-1.28)
HMDB0004824	N2.N2-dimethylguanosine	Nucleosides, nucleotides, and analogues	1.13 (0.97-1.33)	**1.57 (1.08-2.29)**	0.90 (0.60-1.36)
HMDB0005923	N4-acetylcytidine	Nucleosides, nucleotides, and analogues	1.10 (0.96-1.26)	1.26 (0.93-1.70)	1.07 (0.91-1.26)
HMDB0000026	N-carbamoyl-beta-alanine	Ureas	1.09 (0.96-1.25)	**1.43 (1.04-1.97)**	1.02 (0.88-1.18)
HMDB0004949	C16:0 Ceramide	Ceramides	1.07 (0.94-1.22)	1.01 (0.75-1.37)	1.09 (0.94-1.27)
HMDB0011507	C18:2 LPE	Glycerophosphoethanolamines	1.06 (0.93-1.21)	0.95 (0.69-1.32)	1.06 (0.92-1.23)
HMDB0001906	Aminoisobutyric acid	Amino acids, peptides, and analogues	1.05 (0.92-1.19)	0.89 (0.68-1.15)	1.10 (0.95-1.28)
HMDB0000092	Dimethylglycine	Amino acids, peptides, and analogues	1.04 (0.92-1.18)	1.07 (0.79-1.43)	1.04 (0.90-1.19)
HMDB0013713	N-acetyltryptophan	Amino acids, peptides, and analogues	1.04 (0.91-1.18)	1.13 (0.82-1.55)	1.01 (0.88-1.17)
HMDB0011252	C38:4 PC plasmalogen	Glycerophosphocholines	1.01 (0.89-1.15)	0.93 (0.68-1.28)	1.02 (0.88-1.18)
HMDB0000929	Tryptophan	Indolyl carboxylic acids and derivatives	1.01 (0.88-1.16)	1.13 (0.82-1.56)	0.98 (0.84-1.15)
HMDB0029377	Piperine	Alkaloids and derivatives	1.00 (0.88-1.15)	1.03 (0.77-1.39)	0.99 (0.85-1.16)
HMDB0008937	C36:4 PE	Glycerophosphoethanolamines	0.99 (0.87-1.12)	1.09 (0.79-1.50)	0.96 (0.83-1.11)
HMDB0011210	C34:2 PC plasmalogen	NA	0.99 (0.86-1.14)	0.65 (0.44-0.97)	1.06 (0.91-1.24)
HMDB0000688	C5 carnitine	Fatty acid esters	0.99 (0.86-1.13)	1.08 (0.81-1.45)	0.96 (0.83-1.12)
HMDB0000699	1-methylnicotinamide	Pyridinecarboxylic acids and derivatives	0.98 (0.86-1.11)	0.90 (0.66-1.23)	0.99 (0.85-1.15)
HMDB0000884	Ribothymidine	Nucleosides, nucleotides, and analogues	0.97 (0.85-1.11)	0.85 (0.60-1.20)	0.99 (0.85-1.15)
HMDB0002802	Cortisone	Hydroxysteroids	0.97 (0.85-1.11)	0.86 (0.64-1.15)	1.02 (0.87-1.19)
HMDB0001847	Caffeine	Purines and purine derivatives	0.96 (0.85-1.10)	1.01 (0.76-1.35)	0.95 (0.81-1.11)
HMDB0012102	C20:0 SM	Phosphosphingolipids	0.96 (0.85-1.09)	0.97 (0.69-1.34)	0.95 (0.82-1.09)
HMDB0000128	Guanidoacetic acid	Amino acids, peptides, and analogues	0.96 (0.84-1.10)	0.81 (0.61-1.07)	1.01 (0.86-1.18)
HMDB0007883	C34:4 PC	NA	0.96 (0.84-1.09)	1.12 (0.80-1.56)	0.92 (0.80-1.06)
HMDB0009082	C36:2 PE plasmalogen	Glycerophosphoethanolamines	0.95 (0.83-1.08)	**0.72 (0.52-0.99)**	1.00 (0.86-1.15)
HMDB0013130	C5-DC carnitine	Fatty acid esters	0.90 (0.79-1.03)	0.95 (0.68-1.31)	0.89 (0.77-1.03)

All models were conducted using conditional logistic regression that included the matching factors only (age, sex, time of blood collection, race/ethnicity, and menopausal status for women) and further adjusted for BMI, physical activity, regular aspirin use, smoking status, family history of colorectal cancer, endoscopy, alcohol intake, coffee intake, and total calorie intake.

Supplementary Table S4. Association of common metabolites of EDIH and C-peptide biomarker with colorectal cancer risk

HMDB ID	Metabolites	Subclass	HR per 1-SD All participants	HR per 1-SD Men	HR per 1-SD Women
HMDB0000824	C3 carnitine	Fatty acid esters	**1.16 (1.01-1.33)**	1.18 (0.88-1.58)	**1.18 (1.00-1.38)**
HMDB0000301	Urocanic acid	Imidazoles	**1.14 (1.00-1.30)**	1.17 (0.88-1.55)	1.12 (0.96-1.31)
HMDB0003357	N-acetylornithine	Amino acids, peptides, and analogues	1.12 (0.96-1.30)	1.24 (0.86-1.80)	1.09 (0.92-1.29)
HMDB0013331	C14:2 carnitine	Fatty acid esters	1.10 (0.96-1.26)	1.26 (0.93-1.70)	1.07 (0.91-1.26)
HMDB0008942	C38:2 PE	Glycerophosphoethanolamines	1.09 (0.96-1.25)	**1.43 (1.04-1.97)**	1.02 (0.88-1.18)
HMDB0004193	N1-methyl-2-pyridone-5-arboxamide	Pyridinecarboxylic acids and derivatives	1.05 (0.91-1.21)	1.14 (0.84-1.55)	1.00 (0.85-1.18)
HMDB0000161	Alanine	Amino acids, peptides, and analogues	1.04 (0.92-1.18)	1.07 (0.79-1.44)	1.04 (0.90-1.19)
HMDB0029377	Piperine	NA	1.03 (0.90-1.18)	0.74 (0.53-1.02)	1.11 (0.95-1.30)
HMDB0000897	7-methylguanine	Purines and purine derivatives	1.03 (0.90-1.17)	1.12 (0.83-1.53)	1.00 (0.86-1.16)
HMDB0000092	Dimethylglycine	Amino acids, peptides, and analogues	1.03 (0.89-1.18)	1.03 (0.74-1.44)	1.03 (0.89-1.20)
HMDB0000177	Histidine	Amino acids, peptides, and analogues	1.01 (0.89-1.16)	0.92 (0.69-1.23)	1.05 (0.90-1.21)
HMDB0011244	C36:3 PC plasmalogen-A	NA	1.00 (0.88-1.15)	1.03 (0.77-1.39)	0.99 (0.85-1.16)
HMDB0000904	Citrulline	Amino acids, peptides, and analogues	1.00 (0.88-1.14)	0.79 (0.57-1.09)	1.04 (0.90-1.20)
HMDB0005391	C54:6 TAG	Triradylcglycerols	0.99 (0.87-1.14)	0.98 (0.72-1.34)	0.98 (0.84-1.15)
HMDB0001325	Trimethyllysine	Amino acids, peptides, and analogues	0.99 (0.87-1.13)	1.26 (0.94-1.69)	0.92 (0.79-1.08)
HMDB0005923	N4-acetylcytidine	Nucleosides, nucleotides, and analogues	0.97 (0.85-1.11)	0.79 (0.59-1.06)	1.03 (0.88-1.19)
HMDB0000063	Cortisol	Hydroxysteroids	0.94 (0.82-1.08)	1.12 (0.83-1.51)	0.90 (0.77-1.05)
HMDB0001859	Acetaminophen	1-hydroxy-2-unsubstituted benzenoids	0.93 (0.80-1.09)	0.91 (0.64-1.29)	0.95 (0.80-1.14)
HMDB0000641	Glutamine	Amino acids, peptides, and analogues	0.91 (0.79-1.04)	0.80 (0.59-1.09)	0.93 (0.80-1.08)
HMDB0000026	N-carbamoyl-beta-alanine	Ureas	0.90 (0.79-1.04)	1.02 (0.77-1.36)	0.87 (0.74-1.02)
HMDB0000289	Uric acid	Purines and purine derivatives	**0.87 (0.75-1.00)**	**0.74 (0.54-1.01)**	0.92 (0.78-1.08)

All models were conducted using conditional logistic regression that included the matching factors only (age, sex, time of blood collection, race/ethnicity, and menopausal status for women) and further adjusted for BMI, physical activity, regular aspirin use, smoking status, family history of colorectal cancer, endoscopy, alcohol intake, coffee intake, and total calorie intake.
